# Multi-omics analysis identifies distinct subtypes with clinical relevance in lung adenocarcinoma harboring *KEAP1*/*NFE2L2*

**DOI:** 10.7150/jca.66241

**Published:** 2022-02-28

**Authors:** Xiaodong Yang, Ming Li, Zhencong Chen, Xiaobin Fan, Liang Guo, Bo Jin, Yiwei Huang, Qun Wang, Liang Wu, Cheng Zhan

**Affiliations:** 1 Department of Thoracic Surgery, Shanghai Pulmonary Hospital, Tongji University, Shanghai, China.; 2 Department of Thoracic Surgery, Zhongshan Hospital, Fudan University, Shanghai, China.; 3 Department of General Surgery, Xingtai Third Hospital, Hebei Province, China.; 4 Department of Cardiothoracic Surgery, Zhangqiu District People's Hospital, Shandong Province, China.; 5 Department of Thoracic Surgery, Shanghai General Hospital, Shanghai, China.

**Keywords:** lung adenocarcinoma, KEAP1, mutation, NFE2L2

## Abstract

**Backgrounds:** Lung adenocarcinoma is one of the most common malignant tumors, in which *KEAP1*-*NFE2L2* pathway is altered frequently. The biological features and intrinsic heterogeneities of *KEAP1*/*NFE2L2*-mutant lung adenocarcinoma remain unclear.

**Methods:** Multiplatform data from The Cancer Genome Atlas (TCGA) were acquired to identify two subtypes of lung adenocarcinoma harboring *KEAP1*/*NFE2L2* mutations.

Bioinformatic analyses, including immune microenvironment, methylation level and mutational signature, were performed to characterize the intrinsic heterogeneities. Meanwhile, initial results were validated by using in silico assessment of common lung adenocarcinoma cell lines, which revealed consistent features of mutant subtypes. Furthermore, drug sensitivity screening was conducted based on public datasets.

**Results:** Two mutant subtypes (P1 and P2) of 89 patients were identified in TCGA. P2 patients had significantly higher levels of smoking and worse survival compared with P1 patients. The P2 subset was characterized by active immune microenvironment and more smoking-induced genomic alterations with respect to methylation and somatic mutations. Validations of the corresponding features in 20 mutant cell lines were achieved. Several compounds which were sensitive to mutant subtypes of lung adenocarcinoma were identified, such as inhibitors of *PI3K/Akt* and *IGF1R* signaling pathways.

**Conclusions:** *KEAP1*/*NFE2L2-*mutant lung adenocarcinoma showed potential heterogeneities. The intrinsic heterogeneities of *KEAP1*/*NFE2L2* were associated with immune microenvironment and smoking-related genomic aberrations.

## Introduction

Lung cancer is the leading cause of cancer-associated morbidity and mortality worldwide, among which lung adenocarcinoma accounts for the highest proportion with increasing incidence rate 1-7. Previous studies promoted a paradigm shift regarding classifying lung tumors based on the significant genomic alterations for therapeutic targets, such as epidermal growth factor receptor (*EGFR*) and anaplastic lymphoma kinase (*ALK*) 8-10. The Kelch-like ECH-associated protein 1 (*KEAP1*) and the nuclear factor erythroid-2-related factor 2 (*NFE2L2*) mutations were found in more than 20% patients with non-small cell lung cancer, which represented one of the most important genomic subtypes 11,12. Moreover, the genomic alterations of *KEAP1* and *NFE2L2* were reported to play crucial roles in lung adenocarcinoma 13-15.

Abnormal regulations of reactive oxygen species contribute to the occurrence and development of malignant tumors 16. The *KEAP1* and *NFE2L2* are the two main components in the stress response pathways. *KEAP1* mediates the degradation of *NFE2L2* to act as an adaptor protein of the Cullin 3 (*CUL3*) E3 ubiquitin ligase so as to maintain the redox homeostasis. In the presence of oxidative stress, the inactivation of *KEAP1* results in the release, accumulation, and nucleus translocation of *NFE2L2* to counteract the damage 17,18. The *KEAP1*/*NFE2L2* mutations, representing the dysfunctional activations of the stress response pathway, have been found in many malignant tumors, including lung adenocarcinoma 19-21. The *KEAP1*-*NFE2L2* can be hijacked by cancer cells, and the activation of the pathway leads to increased tumor growth and progression 22-24. Nevertheless, the biological features and clinical implications of *KEAP1*/*NFE2L2* mutations remain elusive and contradictory 25. In a retrospective study of 9243 patients (4647 with lung cancer), *KEAP1*/*NFE2L2* mutations were associated with higher tumor mutational burden and higher programmed death-ligand 1 expression. Improved survival was observed in the subset of patients treated with immune checkpoint inhibitors 26. On the contrary, patients with *KEAP1*/*NFE2L2* mutations had inferior survival compared with wild-type patients in subgroup analyses from several trials regarding immunotherapy 27-29. Concurrent mutations with *KEAP1*/*NFE2L2* may also affect patients' benefits from immunotherapy 30,31. Moreover, Hellyer et al suggested that *KEAP1*/*NFE2L2* mutations might represent a mechanism of intrinsic resistance to *EGFR*-tyrosine kinase inhibitor therapy 32. Chemoresistance was also reported to be associated with *KEAP1*/*NFE2L2* mutations 33,34.

In our study, multiplatform data from The Cancer Genome Atlas (TCGA) were acquired to identify two subtypes of lung adenocarcinoma harboring *KEAP1*/*NFE2L2* mutations. Bioinformatic analyses, including immune microenvironment and methylation level, were performed to characterize potential mutant subgroups. The initial results were validated by using in silico assessment of common lung adenocarcinoma cell lines, which revealed consistent features of *KEAP1*/*NFE2L2-*mutant subtypes. Furthermore, cell line samples were adopted for drug sensitivity screening based on public datasets. Potential drugs which were sensitive to each mutant subtype of lung adenocarcinoma were explored.

## Methods

### Patient cohort and cell lines data

First, we selected all patients (565 patients) with primary lung adenocarcinoma in TCGA database. Level 3 RNA sequencing data, DNA methylation data (Illumina Infinium HumanMethylation 450K BeadChip), miRNA expression data and clinical information of patients with lung adenocarcinoma were downloaded from TCGA (https://protal.gdc.cancer.gov/). Somatic mutation data were selected based on previous studies by comprehensive analyses accounting for variance and batch effects 35. Copy number variations (CNV) were estimated using the GISTIC2 method from the University of California Santa Cruz Xena website (https://xena.ucsc.edu). Patients with missing data types in the above were excluded (67 of 565 patients). According to the mutation data, patients with *KEAP1*/*NFE2L2* mutations were selected as the main study cohort (89 patients). The remaining 409 patients without *KEAP1*/*NFE2L2* mutations were regarded as the wild-type group in the subsequent analyses.

RNA sequencing data, miRNA expression levels, copy number values and gene mutation status of common lung adenocarcinoma cell lines were downloaded from the Cancer Cell Line Encyclopedia (CCLE, https://portals.broadinstitute.org/ccle). Also, DNA methylation levels (Illumina Infinium HumanMethylation 450K BeadChip) of selected cancer cell lines were acquired from the Gene Expression Omnibus (GEO, (https://www.ncbi.nlm.nih.gov/geo) (GSE68379). The drug sensitivity data of selected cancer cell lines were obtained from the Genomics of Drug Sensitivity in Cancer (GDSC, https://www.cancerrxgene.org/). Histological information of each cell line was confirmed based on GDSC, CCLE and Cellosaurus database 36,37. Cell lines with unknown data types were removed. In total, 20 lung adenocarcinoma cell lines with *KEAP1*/*NFE2L2* mutations were identified.

### Data processing and clustering

For the DNA methylation data, probes in sex chromosomes or overlapping single nucleotide polymorphisms were removed. Cross-reactive probes were also excluded according to Chen et al 38. The frequencies of six base substitutions (C > A, C > G, C > T, T > A, T > C, and T > G) were calculated. For some datasets, features or probes with more than 20% missing values were deleted. The k-nearest neighbor algorithm was adopted to impute the remaining missing data.

All five data types (RNA sequencing, DNA methylation, miRN, copy number and base substitution) were integrated using the similar network fusion (SNF) method for both lung adenocarcinoma patients and cell lines. The SNF method constructs networks of samples for each available genome-wide data and efficiently fuses them into one network, which represents the full spectrum of underlying features and provides a comprehensive view under a given condition 39. The SNF method has been used and validated in different types of diseases based on multi-omics data 40-43. In this study, the SNF method fused all five datasets into one by creating a similarity matrix for each data type. A non-linear method based on the theory of message-passing was adopted to iteratively update and converge datasets. Afterwards, consensus clustering was performed to identify distinct *KEAP1*/*NFE2L2* mutated subgroups of lung adenocarcinoma patients and cell lines 44.

### Bioinformatic analyses to characterize *KEAP1*/NFE2L2-mutant subgroups

Mutant subgroups were preliminarily characterized by subjecting clusters for both patients and cell lines to Gene Set Enrichment Analysis (GSEA) using Hallmark, Kyoto Encyclopedia of Genes and Genomes (KEGG), and Gene Ontology (GO) (MSigDB v7.0) gene sets 45. Normalized enrichment score >1, nominal *P*-value <0.05, and false discovery rate *Q*-value < 0.25 were used as screening thresholds for GSEA. Moreover, we studied potential concurrent mutations in *KEAP1*/*NFE2L2*-mutant subsets of lung adenocarcinoma patients.

The features of tumor immune microenvironment in *KEAP1*/*NFE2L2*-mutant lung adenocarcinoma were evaluated according to several previous studies. Saltz et al proposed a leukocyte fraction by estimating tumor-infiltrating leukocytes on hematoxylin and eosin stained slides using deep learning techniques 46. We also used the “Estimation of STromal and Immune cells in MAlignant Tumours using Expression data (ESTIMATE)” method for the assessment of tumor immune microenvironment 47. Li et al developed a public resource (Tumor IMmune Estimation Resource, TIMER) to study tumor-infiltrating immune cells by computational approaches based on RNA sequencing 48. The levels of specific immune cell infiltration, like CD8+ T cell and macrophage, between mutant subgroups were compared. Furthermore, we compared the number of immunogenic mutations per sample stratified by the *KEAP1*/*NFE2L2* mutant status.

The global methylation levels (β value) between *KEAP1*/*NFE2L2-*mutant patient subgroups and cell line subsets were compared to investigate epigenomic alterations and potential clinical associations. Next, a list of smoking-related DNA methylation probes was obtained from a previous study conducted by Vaz et al. Vaz et al performed two repeated experiments with respect to chronic-cigarette-smoking-induced hypermethylated probes 49.The union of all reported probes was extracted and their levels stratified by the mutant subsets were compared. Somatic mutation status of *KEAP1/NFE2L2*-mutant patients was analyzed to extract mutational signatures using the SignatureAnalyzer 50. Similarities were studied based on previously reported thirty mutational signatures in the Catalogue Of Somatic Mutations In Cancer (COSMIC, https://cancer.sanger.ac.uk/cosmic) to identify the potential clinical associations and etiologies.

Cancer-associated drug sensitivity data of lung adenocarcinoma cell lines were also downloaded from two sub-datasets of GDSC. Drug samples that were tested in < 50% cell lines were excluded. The natural log value of the fitted half-maximal inhibitory concentration [LN(IC50)] of each drug was adopted to select caner-associated drugs which were specifically sensitive to mutant subtypes (C1 and C2). Many attempts have been made to perform *in vitro* pharmacogenomic response analyses based on the publicly available GDSC datasets 51-53. The parameter IC50 was also adopted in previous studies 54-56. The criteria for *KEAP1*/*NFE2L2*-mutant specific drugs were as follows: LN(IC50)_C1 or C2_ < LN(IC50)_C2 or C1_, *P* < 0.05; LN(IC50)_C1 or C2_ < LN(IC50)_WT_, *P* < 0.05; and LN(IC50)_C2 or C1_ ≈ LN(IC50)_WT_, *P* > 0.05. However, only one C2-specific drug could be identified using the revised criteria: LN(IC50)_C2_ < LN(IC50)_C1_, *P* < 0.1; LN(IC50)_C2_ < LN(IC50)_WT_, *P* < 0.1; and LN(IC50)_C1_ ≈ LN(IC50)_WT_, *P* > 0.1.

### Statistical analysis

All statistical analyses in this study were conducted using R version 3.6.1 (R Foundation for Statistical Computing, Vienna, Austria) and IBM SPSS Statistics 22.0 (IBM, Inc., NY, USA). Comparisons of immunological features and drug sensitivities were performed using the Kruskal-Wallis H test and Mann-Whitney U test. Baseline characteristics and co-mutations were studied by the chi-square test. Survival curves were estimated and compared following the Kaplan-Meier method and the log-rank test. A two-tailed *P*-value less than 0.05 was considered statistically significant.

## Results

### Identification of subtypes of *KEAP1*/*NFE2L2*-mutant lung adenocarcinoma

**Figure 1:**
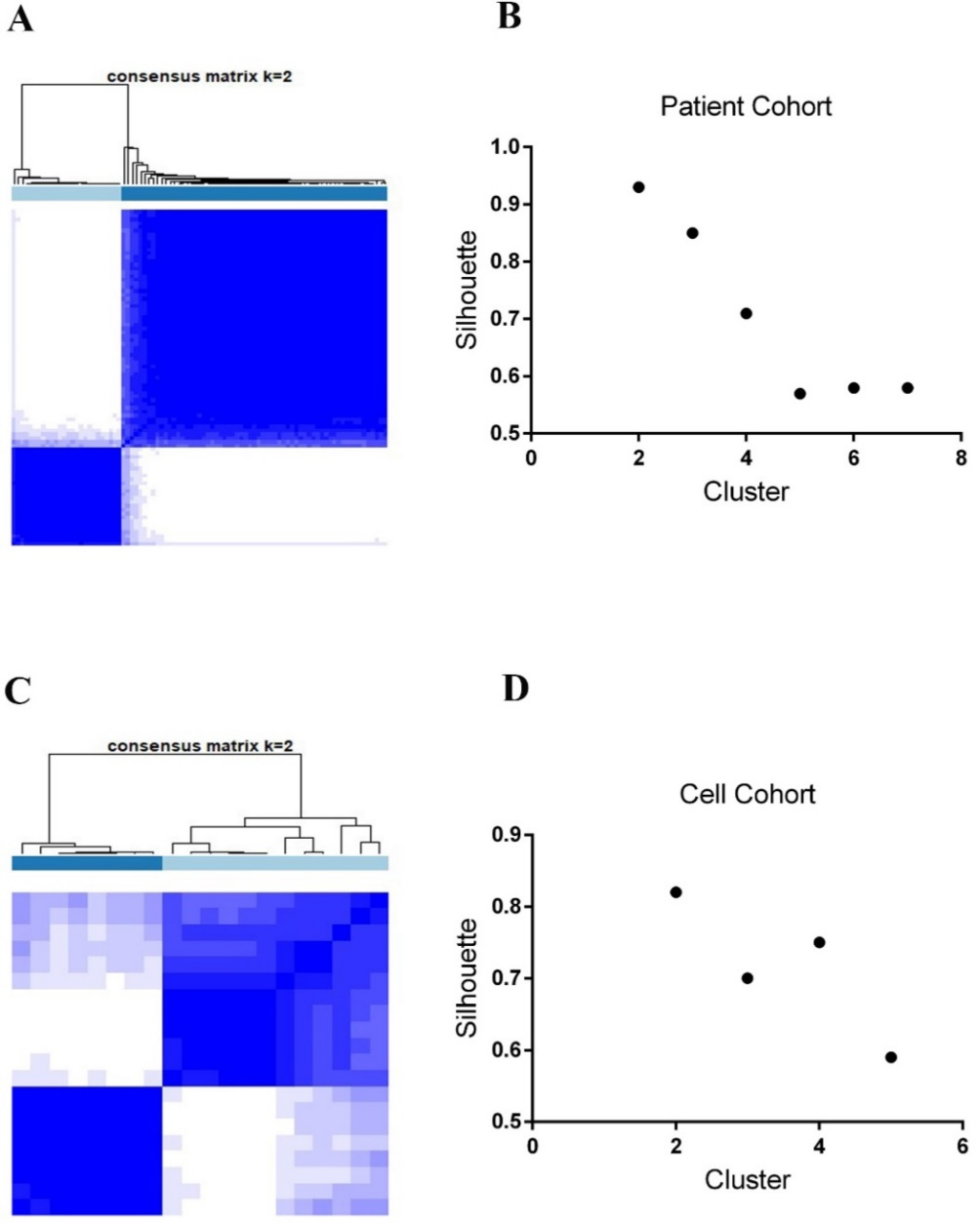
** The SNF fused five types of datasets and consensus clustering identifies subsets of *KEAP1*/*NFE2L2*-mutant lung adenocarcinoma in patients and cell lines. A.** Two subsets of *KEAP1*/*NFE2L2*-mutant patients were identified. **B.** Silhouette values of patient clustering with the k = 2 to 7. **C.** Two subsets of *KEAP1*/*NFE2L2*-mutant cell lines were identified. **D.** Silhouette values of cell line clustering with the k = 2 to 5.

As previously stated in the Methods section, we integrated five data subtypes and clustered 89 *KEAP1/NFE2L2*-mutant lung adenocarcinoma patients into two subgroups (P1 and P2 groups, Figure 1A). Similarly, two subtypes were identified in 20 lung adenocarcinoma cell lines harboring *KEAP1*/*NFE2L2-*mutations (C1 and C2 groups, Figure 1C). Clustering with two classes in both patients and cell line samples showed the highest silhouette values (silhouette = 0.93 and 0.83, Figure 1B and 1D).

### Clinicopathological differences of the *KEAP1*/*NFE2L2*-mutant subtypes

**Figure 2:**
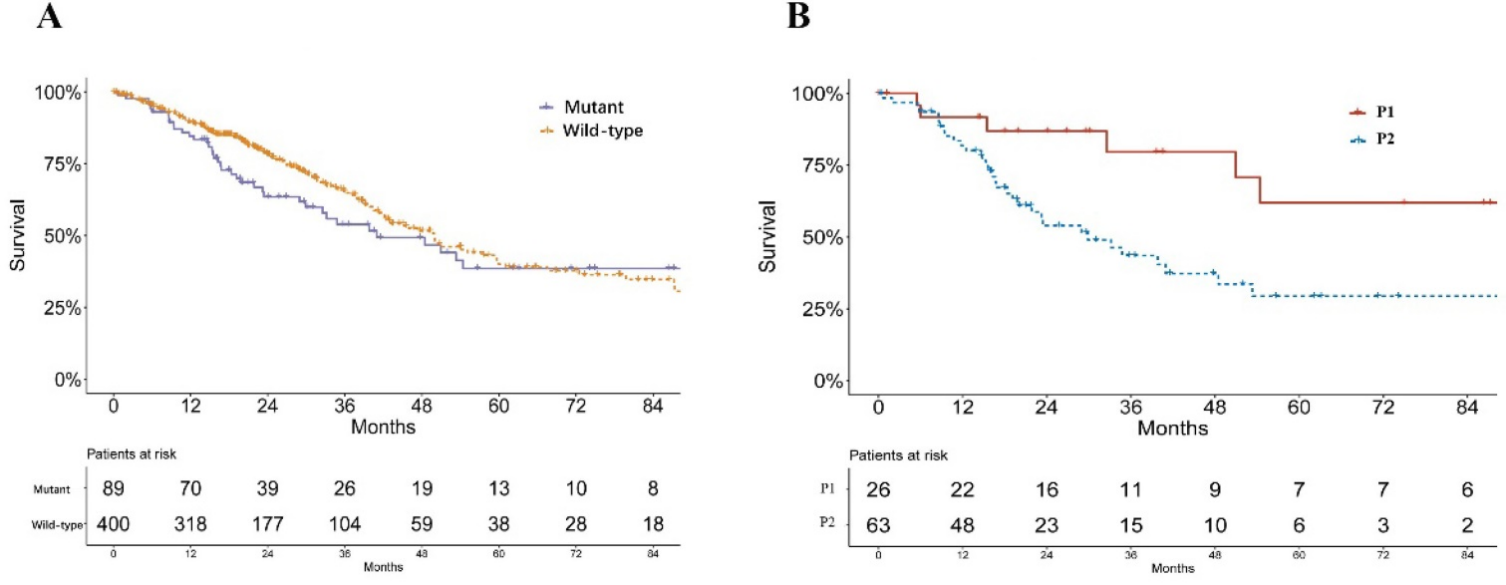
** Survival curves of lung adenocarcinoma patients in TCGA. A.** Survival curves of *KEAP1*/*NFE2L2*-mutant and wild-type patients (*P* = 0.212). **B.** Survival curves of *KEAP1*/*NFE2L2*-mutant patient subgroups (P1 and P2) (*P* = 0.020).

A significant difference was found in the smoking status of patients among P1, P2 and wild-type groups (*P* = 0.033, Table 1). The P2 group consisted of the highest proportions of current smokers and reformed smokers for ≤15 years, while P1 groups consisted of more reformed smokers ≥ 15 years (Table 1). No significant difference of pathological stage was found among patients of P1, P2 and *KEAP1*/*NFE2L2* wild-type lung adenocarcinoma (*P* = 0.233, Table 1). Mutant samples contained a significantly higher proportion of female patients (*P* = 0.003, Table 1). Survival analysis showed no significant difference in overall survival between subgroups of *KEAP*/*NFE2L2-*mutant and wild-type lung adenocarcinoma (*P* = 0.212, Figure 2A). However, the P2-mutant subgroup was associated with a significantly worse survival than the P1 subgroup (*P* = 0.020, Figure 2B).

### Basic biological features of *KEAP1*/*NFE2L2*-mutant subtypes

**Figure 3:**
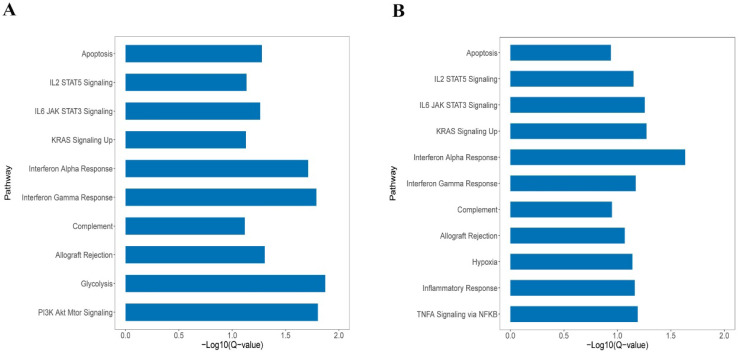
** A.** The enriched pathways in Hallmark of *KEAP1*/*NFE2L2*-mutant P2 patient subgroup. **B.** The enriched pathways in Hallmark of *KEAP1*/*NFE2L2*-mutant C2 cell line subgroup.

GSEA was performed in *KEAP1*/*NFE2L2-*mutant subtypes in both patients and cell line cohorts. As shown in Figure 3A and 3B, the P2 and C2 subtypes were both enriched in the same pathways, such as *KRAS* signaling, *IL2*/*STAT5* signaling, apoptosis, and interferon alpha and gamma response. GSEA revealed similarities between the P2 and C2 subtypes, validating the integration and clustering process to some degree.

Moreover, both P2 and C2 subtypes were associated with regulations of immune-related pathways, such as activations of T cells and macrophages (Supplement Figure 1A and 1B). The results revealed that the P2 and C2 subgroups displayed active immune pathways compared with P1 and C1 subgroups, respectively.

The P2 subgroup was found associated with higher proportions of *TP53* (*P* < 0.001), *PCLO* (*P* = 0.011), *NF1* (*P* = 0.029) and *PTPRT* (*P* = 0.040) mutations, while the P1 subgroup may have more patients with *STK11* (*P* = 0.008) mutation (Supplement Table 1). However, we did not validate the mutational associations in lung adenocarcinoma cell lines due to the small sample size.

### Immunological features of the *KEAP1*/*NFE2L2*-mutant subtypes

**Figure 4:**
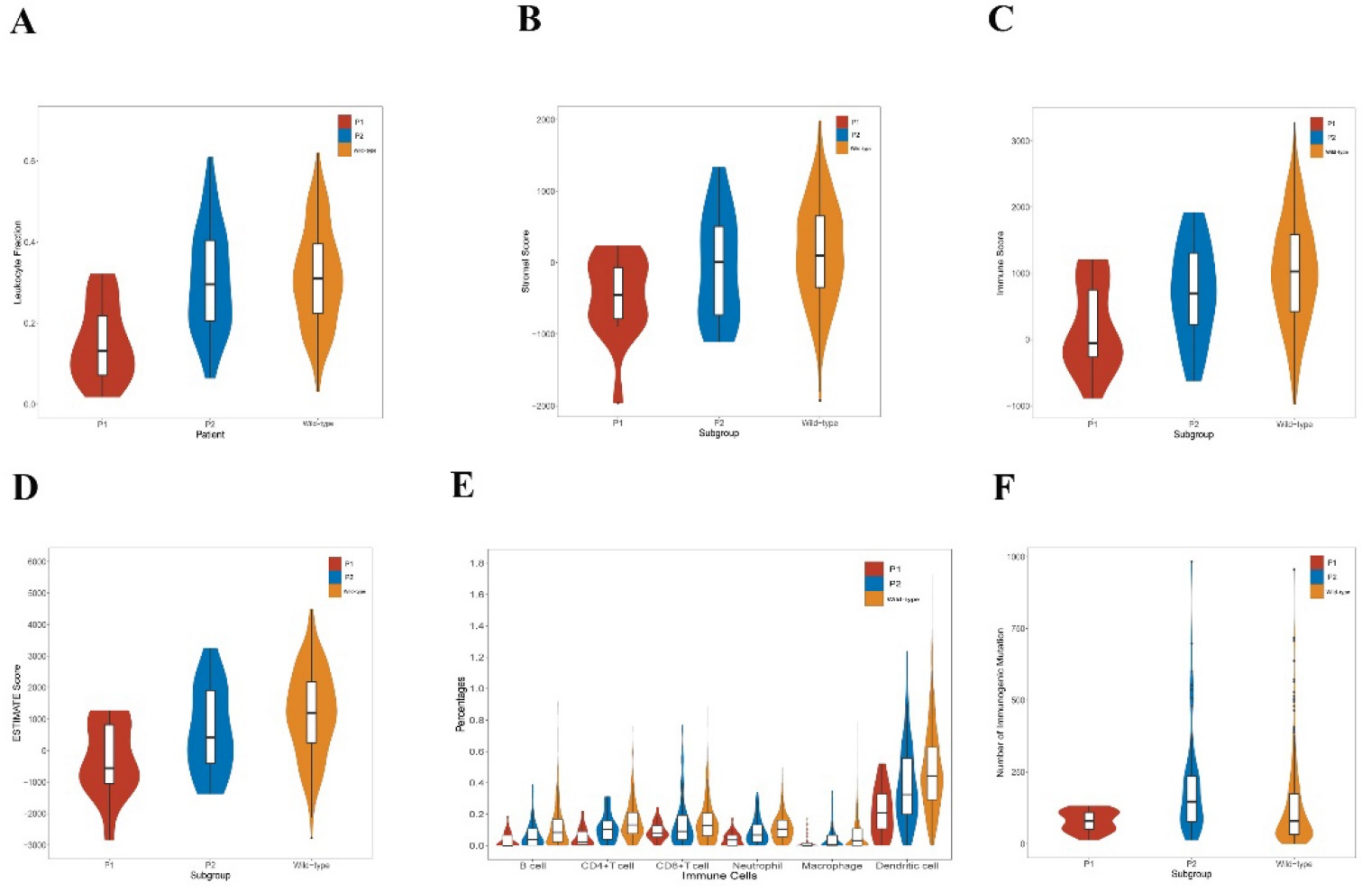
** Immunological features of lung adenocarcinoma patients in TCGA. A.** Comparison of leukocyte fraction stratified by *KEAP1*/*NFE2L2*-mutant (P1 and P2) and wild-type patient subgroups (mutant group vs. wild-type group, *P* = 0.001; P1 group vs. P2 group, P < 0.001). **B.** Comparison of stromal score calculated by ESTIMATE algorithm stratified by *KEAP1*/*NFE2L2*-mutant (P1 and P2) and wild-type patient subgroups (P1 vs. P2 vs. wild-type group, *P* = 0.005). **C.** Comparison of immune score calculated by ESTIMATE algorithm stratified by *KEAP1*/*NFE2L2*-mutant (P1 and P2) and wild-type patient subgroups (P1 vs. P2 vs. mutant group, *P* = 0.001). **D.** Comparison of ESTIMATE score calculated by ESTIMATE algorithm stratified by *KEAP1*/*NFE2L2*-mutant. (P1 and P2) and wild-type patient subgroups (P1 vs. P2 vs. wild-type group, *P* = 0.001). **E.** Comparison of tumor-infiltrating immune cells stratified by *KEAP1*/*NFE2L2*-mutant (P1 and P2) and wild-type patient subgroups based on TIMER database. [mutant group vs. wild-type group: CD4+ T cells (*P* < 0.001), CD8+ T cells (*P* = 0.011), B cells (*P* < 0.001), neutrophils (*P* < 0.001), dendritic cells (*P* < 0.001), and macrophages (*P* = 0.008); P1 group vs. P2 group: B cells (*P* = 0.017), CD4+ T cells (*P* = 0.001), CD8+ T cells (*P* = 0.375), neutrophils (*P* = 0.002), macrophages (*P* = 0.113), and dendritic cells (*P* = 0.006)]. **F.** Comparison of the number of immunogenic mutations per sample stratified by *KEAP1*/*NFE2L2*-mutant (P1 and P2) and wild-type patient subgroups (P1 vs. P2 vs wild-type group, P < 0.001).

The tumor-infiltrating lymphocyte fractions were compared according to Saltz et al stratified by the mutation status 46. Compared with the wild-type samples, lung adenocarcinoma harboring *KEAP1*/*NFE2L2* had a significantly lower lymphocyte fractions (*P* = 0.001, Figure 4A). Subgroup analyses revealed that the P2 group exhibited significantly higher lymphocyte fractions compared with the P1 group (*P* < 0.001, Figure 4A). We also observed that significant differences of ESTIMATE scores exist among three groups, in which P1 was related to the lowest score (Figure 4B, 4C and 4D). Based on TIMER, a significant decrease was found in the infiltrating levels of CD4+ T cells (*P* < 0.001), CD8+ T cells (*P* = 0.011), B cells (*P* < 0.001), neutrophils (*P* < 0.001), dendritic cells (*P* < 0.001), and macrophages (*P* = 0.008) in the mutant subgroup (Figure 3B). Moreover, the P1 subgroup was associated with reduced infiltrations of B cells (*P* = 0.017), CD4+ T cells (*P* = 0.001), neutrophils (*P* = 0.002) and dendritic cells (*P* = 0.006) (Figure 4E). Furthermore, the P2 subtype was associated with higher number of immunogenic mutations than the P1 group (Figure 4F).

### Smoking-related genomic features of the *KEAP1*/*NFE2L2*-mutant subtypes of lung adenocarcinoma

**Figure 5:**
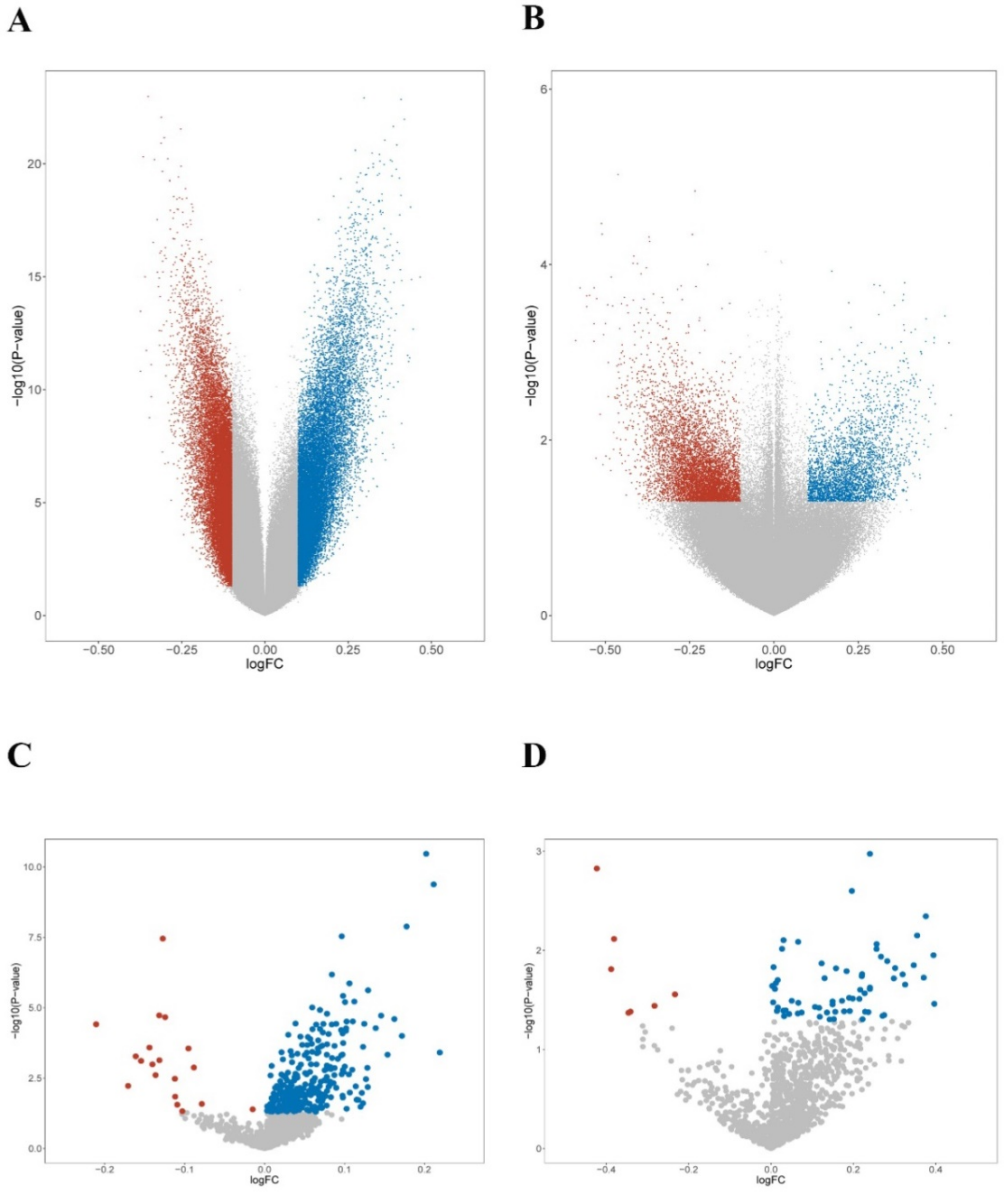
** Epigenomic features of *KEAP1*/*NFE2L2*-mutant subgroups of lung adenocarcinoma patients and cell lines. A.** Volcano plot of the global DNA methylation difference between patient mutant subgroups (P1 and P2). **B.** Volcano plot of the global DNA methylation difference between cell line mutant subgroups (C1 and C2). **C.** Volcano plot of the smoking-related methylation signatures between patient mutant subgroups (P1 and P2). **D.** Volcano plot of the smoking-related methylation signatures between cell line mutant subgroups (C1 and C2).

First, the methylation levels were compared across mutant subgroups. 84,700 and 64,204 differentially hypermethylated probes were found in the P1 and P2 groups, respectively (Figure 5A). Meanwhile, 8,981 hypermethylated probes were found in the C1 group, while 5,933 hypermethylated probes were found in the C2 group (Figure 5B). Next, unique smoking-related probes were extracted according to Vaz et al 49. Both P2 and C2 groups displayed a similar trend of hypermethylation compared with the P1 and C1 groups (Figure 5C-D). The results suggested that smoking-related epigenomic alterations might play essential roles in *KEAP1*/*NFE2L2-*mutant subgroups. The epigenomic similarities confirmed a potential resemblance between patient and cell line mutant subsets.

Second, we assessed the somatic mutational patterns of all lung adenocarcinoma patients and obtained four distinctive signatures (Supplement Figure 2A). Among them, signature 2 subgroup (W2) was like Signature 4 and 29 of the thirty known somatic mutational signatures in the COSMIC database, which were closely associated with smoking and tobacco chewing (coefficient of cosine similarity = 0.805 and 0.740). Then, we compared the normalized activities of the identified W2 mutational signature between *KEAP1*/*NFE2L2*-mutant subgroups. We found that the P2 subset had significantly higher activities of W2 signature than the P1 subset (Supplement Figure 2B, *P* = 0.004), which further indicated possible different roles of smoking in the mutant subgroups.

### Screening for compounds with potential sensitivity to the *KEAP1*/*NFE2L2*-mutant subtypes

After characterizing the clinical and biological features of the mutant subtypes, possible cancer-associated drugs which were sensitive to each subtype were explored. More than 400 drugs and compounds were tested on *KEAP1*/*NFE2L2*-mutant and wild-type lung adenocarcinoma cell lines in GDSC. This part aimed to target cancer-associated drugs and compounds with potential specific sensitivity to the C1 or C2 subset. 38 drugs, which were potentially sensitive to the C2 mutant subtype, were discovered (Supplement Table 2). Although the criteria were adjusted, only one C1-specific compound was identified (Supplement Table 2).

**Figure 6:**
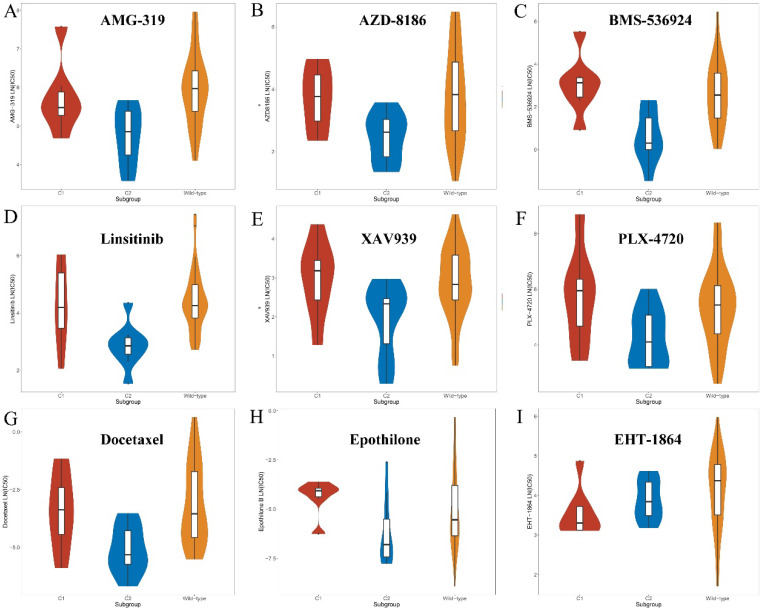
** Screened drugs with selective sensitivity toward the *KEAP1*/*NFE2L2*-mutant subtypes. A-H.** Drugs that selectively killed tumor cells of the C2 subset. **I.** Drug that selectively killed tumor cells of the C1 subset.

C2-specific drugs were found to be mainly composed of the following types. First, inhibitors of the *PI3K*/*Akt* signaling pathways, such as afuresertib, AZD8186 and AMG-319 might be sensitive to the C2 subgroup compared with the C1 and wild-type groups (Figure 6 and Supplement Table 2). Second, inhibitors of *IGF1R* signaling, such as BMS-536924, linsitinib and NVP-ADW742, showed better efficacy in the C2 subset (Figure 6 and Supplement Table 2). Moreover, drugs that target *Wnt* and *MAPK*/*Erk* signaling pathways were more toxic to the C2 subgroup (Figure 6 and Supplement Table 2). In addition, chemotherapy drugs, such as docetaxel, epothilone B and vinorelbine were found to preferentially kill tumor cells of the C2 subgroup (Figure 6 and Supplement Table 2). Nevertheless, only one compound (EHT-1864) was found that might be sensitive to the C1 subset (Figure 6 and Supplement Table 2). The selected compound, EHT-1864, is an inhibitor of *Rac1*, *Rac2* and *Rac3* and mediated the reorganization of actin cytoskeleton.

## Discussion

The *KEAP1*/*NFE2L2* mutations were observed in many common malignant tumors, including lung adenocarcinoma 11,12,19,21, which might define a molecular subset of rapidly progressing tumor 57. In this study, the multiplatform data from TCGA were adopted to identify subsets of lung adenocarcinoma with *KEAP1*/*NFE2L2* mutations. Clinicopathological and bioinformatics analyses, such as immune microenvironment and methylation level, were performed to further explore the intrinsic heterogeneities of *KEAP1*/*NEFE2L2*-mutant disease. Moreover, cell line samples were used for drug sensitivity screening based on public datasets. In addition, *CUL3* mutation was not included as the genomic signature in this study. *CUL3* belonged to the ubiquitin-proteasome system, which was involved in many oncogenic processes, and could not be considered as a specific *KEAP1*/*NFE2L2* pathway component 58.

Variations in the *KEAP1*-*NFE2L2* pathway were detected in more than 20% patients with lung cancer, which represented one of the major molecular subtypes 11,12. Goeman et al revealed that *KEAP1*/*NFE2L2* mutations represented a negative factor of survival, which defined a rapidly progressing molecular subtype 57,59. The mutant type showed heterogeneities, and one subset was associated with significantly worse survival. Cai et al performed a similar study and divided *KEAP1*/*NFE2L2*-mutant lung adenocarcinoma into three subsets based on gene profiling. The present study integrated multi-omics datasets, such as somatic mutation, methylation, and miRNA, to cluster into two subsets. P2/C2 subset displayed active immune pathways compared with the P1/C1 subgroups. The controversies of the prognosis regarding patients with *KEAP1/NFE2L2* mutations treated with immunotherapy may be associated with the distinct immune microenvironment of P1 and P2 subgroups 25. Ricciuti B et al revealed that lung adenocarcinoma harboring concurrent *KRAS*/*STK11* and *KRAS*/*KEAP1* mutations display distinct immune profiles 30. In this work, we also observed different patterns of concurrent mutations between mutant subsets. Clinical features, somatic mutation signatures and methylation levels showed potential associations with patients' smoking history. Previous studies demonstrated that smoking led to significant nuclear translocation of *NFE2L2,* which might be potentially fatal in smoking-related lung tumorigenesis 60,61. These findings might also be potential evidence of distinct *KEAP1*/*NFE2L2* subtypes.

Furthermore, drug sensitivities of cell lines from public datasets were analyzed and several subgroup-specific drugs were discovered in our study. Best et al observed that synergy between *KEAP1*/*NFE2L2* and *PI3K* pathways promoted lung cancer progression with the altered immune milieu, which supported the compound screening results of inhibitors of *PI3K*/*Akt* pathways in this study 13. Several studies revealed possible associations between the two pathways 62,63. The pathway analyses of this study also revealed that *PI3K*/*Akt* pathway was enriched in the P2 subgroup. Vartanian et al identified alternative pathways critical for *NFE2L2*-dependent growth in *KEAP1*-mutant cell lines, including *IGF1R* 64. The findings in this study suggested that inhibitors of *IGF1R* signaling were effective in the C2 subtype. Only one alternative compound existed, which inhibited *Rac* signaling to mediate the actin cytoskeleton. Wu et al demonstrated that *KEAP1* stabilized F-actin cytoskeleton structures and inhibited focal adhesion, thereby restraining migrations and invasions of lung cancers 65. *KEAP1*/*NFE2L2*/*CUL3* represented a mechanism of resistance to tyrosine kinase inhibitor in patients with *EGFR*-mutant non-small cell lung cancer 32. Most identified compounds in our study were sensitive to the C2 subgroup which represented a subset with a worse prognosis. However, only one compound showed better efficacy to the C1 group with a revised statistical threshold, revealing difficulties in selecting appropriate drugs. However, the intrinsic differences in immune infiltrations suggested distinct immunotherapy strategies, especially developing drugs for the C2/P2 group. Also, concurrent alterations, like *STK11* and *TP53*, could also be potential targets in *KEAP1*/*NFE2L2*-mutant diseases.

There were also limitations that should be mentioned in this study. First, it had a small sample size of mutant cell lines and patients. The study explored intrinsic heterogeneities of *KEAP1*/*NFE2L2*-mutant lung adenocarcinoma. However, further studies are required to better characterize and precisely differentiate each mutant subtype. Although LN(IC50) was adopted from GDSC to measure compound sensitivities, more experiments should be conducted to test drug efficacy.

## Conclusion

Two subtypes of *KEAP1*/*NFE2L2*-mutant lung adenocarcinoma were identified based on both patient and cell line samples, and genomic and clinicopathological features of *KEAP1*/*NFE2L2* mutations were characterized. The intrinsic heterogeneities of *KEAP1*/*NFE2L2* mutations was found to be associated with immune microenvironment and smoking-related genomic aberrations.

## Supplementary Material

Supplementary figures and tables.

## Figures and Tables

**Table 1:** Baseline characteristics of wild type and *KEAP1/NFE2L2*-mutant subgroups of patients with lung adenocarcinoma in TCGA

	Wild type	Mutant P1 group	Mutant P2 group	*P*-value
**Age**	65.3 ± 9.9	67.6 ± 7.1	64.3 ± 11.2	
**Gender**				0.003
Female	234 (57.2)	12 (46.2)	22 (34.9)	
Male	175 (42.8)	14 (53.8)	41 (65.1)	
**Pathological Stage**				0.233*
Stage I	226 (55.3)	14 (53.8)	30 (47.6)	
Stage II	99 (24.2)	5 (19.2)	17 (27.0)	
Stage III	67 (16.4)	5 (19.2)	9 (14.3)	
Stage IV	15 (3.7)	2 (7.7)	7 (11.1)	
Unknown	2 (0.5)	0 (0)	0 (0)	
**Smoking Status**				0.033*
Non-smoker	66 (16.1)	1 (3.8)	5 (7.9)	
Current smoker	96 (23.5)	5 (19.2)	16 (25.4)	
Reformed smoker (> 15 years)	105 (25.7)	12 (46.2)	11 (17.5)	
Reformed smoker (≤ 15 years)	127 (31.1)	8 (30.8)	28 (44.4)	
Unknown	15 (3.7)	0 (0)	3 (4.8)	

* Samples with unknown information were removed when comparisons were conducted among groups.
