# Supplementary material for: Multi-omics analysis identifies distinct subtypes with clinical relevance in lung adenocarcinoma harboring KEAP1/NFE2L2
Source: J Cancer. 2022 Feb 28;13(5):1512–22. doi: 10.7150/jca.66241 (PMC8965119; doi:10.7150/jca.66241)
Supplement: Supplementary file 1 — Supplementary figures and tables. [file jcav13p1512s1.pdf]

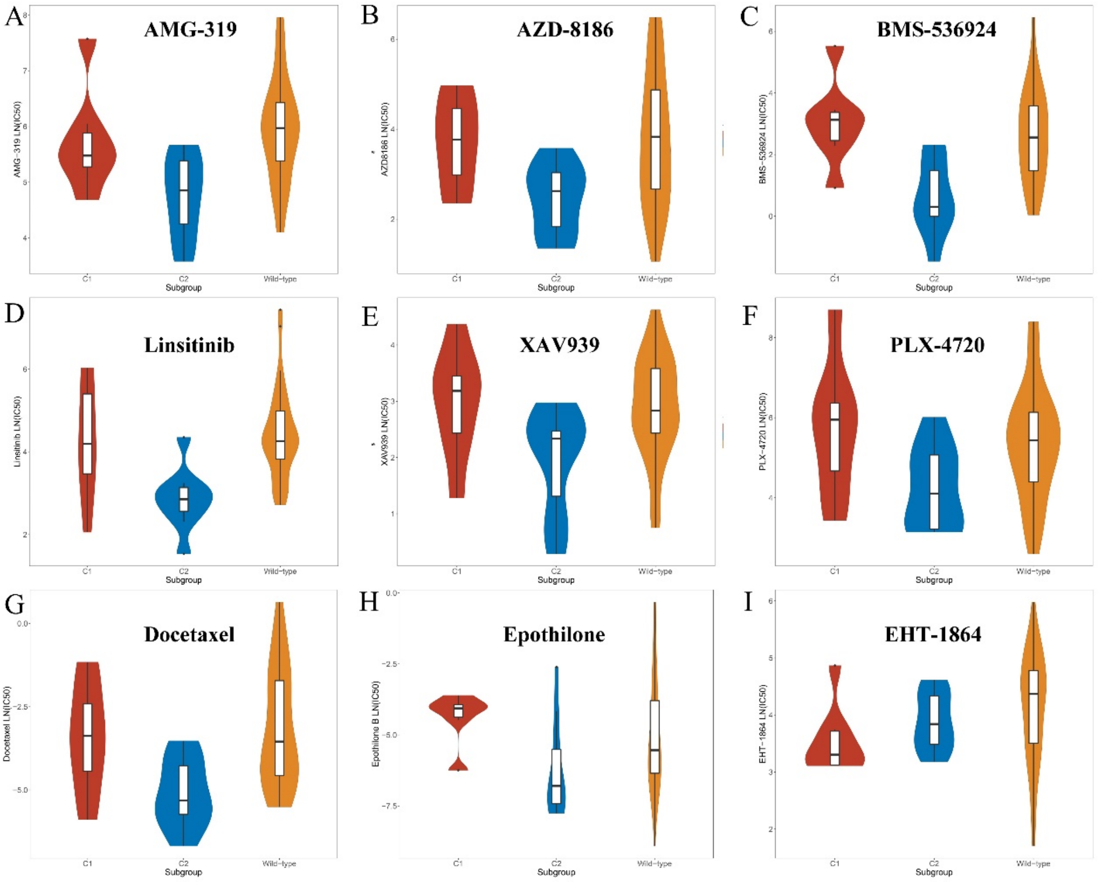

Supplement Figure 1:

A. The enriched immune-related pathways in GO of *KEAP1*/*NFE2L2*-mutant P2 patient subgroup.

B. The enriched immune-related pathways inGO of *KEAP1*/*NFE2L2*-mutant C2 patient subgroup.

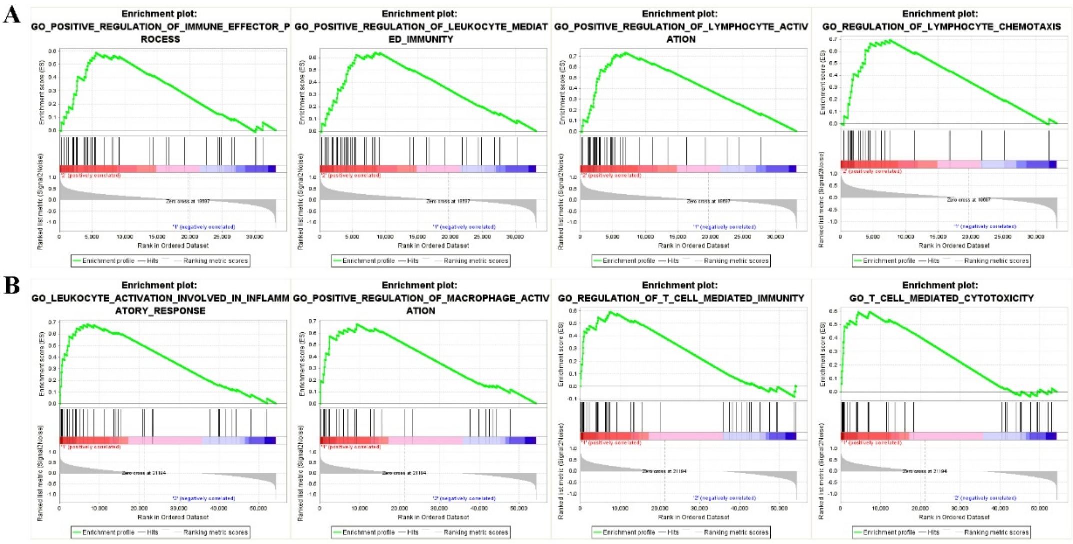

Supplement Figure 2:

A. Four subgroups with distinct mutational signatures were retrieved from the mutational profiles of TCGA lung adenocarcinoma patients. W2 subgroup was closely associated with smoking-related mutational signatures.

B. Differential activities of the smoking-related mutational signature between the subtypes of *KEAP1*/*NFE2L2*-mutant lung adenocarcinoma patients (P = 0.004).

Supplement Table 1: Comparisons of common mutations between *KEAP1/NFE2L2*-mutant subgroups of patients (P1 and P2).

	P1	P2	*P*-value
Number	26	63	
EGFR mutation			1.000*
Yes	1 (3.8)	2 (3.2)	
No	25 (96.2)	61 (96.8)	
KRAS mutation			0.081
Yes	10 (38.5)	13 (20.6)	
No	16 (61.5)	50 (79.4)	
TP53 mutation			<0.001*
Yes	3 (11.5)	37 (58.7)	
No	23 (88.5)	26 (41.3)	
STK11 mutation			0.008
Yes	11 (42.3)	10 (15.9)	
No	15 (57.7)	53 (84.1)	
LRP1B mutation			0.113*
Yes	4 (15.4)	22 (34.9)	
No	22 (84.6)	41 (64.1)	
PCLO mutation			0.011*
Yes	1 (3.8)	20 (31.7)	
No	25 (96.2)	43 (68.3)	
PTPRD mutation			0.756*
Yes	2 (7.7)	8 (12.7)	
No	24 (92.3)	55 (87.3)	
RELN mutation			0.550*
Yes	4 (15.4)	15 (23.8)	
No	22 (84.6)	48 (76.2)	
FAT4 mutation			0.476*
Yes	3 (11.5)	13 (20.6)	
No	23 (88.5)	50 (79.4)	
KMT2D mutation			0.316#
Yes	0 (0)	5 (7.9)	
No	26 (100)	58 (92.1)	
EPHA5 mutation			0.129*
Yes	1 (3.8)	12 (19.0)	
No	25 (96.2)	51 (81.0)	
CPS1 mutation			0.920*
Yes	2 (7.7)	7 (11.1)	
No	24 (92.3)	56 (88.9)	
NF1 mutation			0.029#

Yes	0 (0)	11 (17.5)	
No	26 (100)	52 (82.5)	
SETBP1 mutation			1.000*
Yes	4 (15.4)	9 (14.3)	
No	22 (84.6)	54 (85.7)	
FAT1 mutation			0.613*
Yes	2 (7.7)	9 (14.3)	
No	24 (92.3)	54 (85.7)	
PTPRT mutation			0.040*
Yes	1 (3.8)	16 (25.4)	
No	25 (96.2)	47 (74.6)	
GRIN2A mutation			1.000*
Yes	2 (7.7)	5 (7.9)	
No	24 (92.3)	58 (92.1)	
ZNF521 mutation			0.054#
Yes	0 (0)	9 (14.3)	
No	26 (100)	54 (85.7)	
MGAM mutation			0.175#
Yes	0 (0)	6 (9.5)	
No	26 (100)	57 (90/5)	

All comparisons were based on the chi-square test.

* represents the chi-square test with continuity correction.

# represents the Fisher's exact test.

Supplement Table 2: The list of identified mutant-specific compounds based on the GDSC database.

GDSC database	Drug ID	Drug Name	Putative Target	Pathway Name	Subgroup Specific
GDSC2	1576	IWP-2	PORCN	WNT signaling	C2
GDSC2	1854	MN-64	TNKS1, TNKS2	WNT signaling	C2
GDSC1	1268	XAV939	TNKS1, TNKS2	WNT signaling	C2
GDSC2	1268	XAV939	TNKS1, TNKS2	WNT signaling	C2
GDSC1	308	Foretinib	MET, KDR, TIE2, VEGFR3/FLT4, RON, PDGFR, FGFR1, EGFR	RTK signaling	C2
GDSC1	1029	Motesanib	VEGFR, RET, KIT, PDGFR	RTK signaling	C2
GDSC1	1049	PD173074	FGFR1, FGFR3	RTK signaling	C2
GDSC1	1194	SB505124	TGFBR1, ACVR1B, ACVR1C	RTK signaling	C2
GDSC2	1912	Afuresertib	AKT1, AKT2, AKT3	PI3K/MTOR signaling	C2
GDSC1	228	AKT inhibitor VIII	AKT1, AKT2, AKT3	PI3K/MTOR signaling	C2
GDSC2	2045	AMG-319	PI3Kalpha, PI3Kdelta, PI3Kgamma	PI3K/MTOR signaling	C2
GDSC2	1918	AZD8186	PI3Kalpha, PI3Kbeta	PI3K/MTOR signaling	C2
GDSC2	1053	MK-2206	AKT1, AKT2	PI3K/MTOR signaling	C2
GDSC1	1527	Pictilisib	PI3K (class 1)	PI3K/MTOR signaling	C2
GDSC1	223	ZSTK474	PI3K (class 1)	PI3K/MTOR signaling	C2
GDSC2	1916	AZD5363	AKT1, AKT2, AKT3, ROCK2	Other, kinases	C2
GDSC1	229	Enzastaurin	PKCB	Other, kinases	C2
GDSC1	253	XMD14-99	ALK, CDK7, LTK, others	Other, kinases	C2
GDSC1	332	XMD15-27	CAMK2	Other, kinases	C2
GDSC1	186	Bexarotene	Retinioic X receptor (RXR) agonist	Other	C2
GDSC2	1998	BPD-00008900		Other	C2
GDSC1	166	FTI-277	Farnesyl-transferase (FNTA)	Other	C2
GDSC1	180	Thapsigargin	SERCA	Other	C2
GDSC2	1007	Docetaxel	Microtubule stabiliser	Mitosis	C2

GDSC1	201	Epothilone B	Microtubule stabiliser	Mitosis	C2
GDSC1	225	Genentech Cpd 10	AURKA, AURKB	Mitosis	C2
GDSC1	140	Vinorelbine	Microtubule destabiliser	Mitosis	C2
GDSC2	1913	AGI-5198	IDH1 (R132H)	Metabolism	C2
GDSC2	1091	BMS-536924	IGF1R, IR	IGF1R signaling	C2
GDSC2	1510	Linsitinib	IGF1R	IGF1R signaling	C2
GDSC2	1932	NVP-ADW742	IGF1R	IGF1R signaling	C2
GDSC2	1036	PLX-4720	BRAF	ERK MAPK signaling	C2
GDSC1	1061	SB590885	BRAF	ERK MAPK signaling	C2
GDSC1	1069	EHT-1864	RAC1, RAC2, RAC3	Cytoskeleton	C1
GDSC2	1928	I-BRD9	BRD9	Chromatin other	C2
GDSC2	1237	EPZ004777	DOT1L	Chromatin histone methylation	C2
GDSC2	1563	EPZ5676	DOT1L	Chromatin histone methylation	C2
GDSC1	271	VNLG/124	HDAC,RAR	Chromatin histone acetylation	C2
GDSC2	1997	WEHI-539	BCL-XL	Apoptosis regulation	C2
